# Expression of PD-1 Molecule on Regulatory T Lymphocytes in Patients with Insulin-Dependent Diabetes Mellitus

**DOI:** 10.3390/ijms160922584

**Published:** 2015-09-18

**Authors:** Valentina Perri, Benedetta Russo, Antonino Crinò, Riccardo Schiaffini, Ezio Giorda, Marco Cappa, Maria Manuela Rosado, Alessandra Fierabracci

**Affiliations:** 1 Immunology and Pharmacotherapy Area, Children’s Hospital Bambino Gesù, Viale S. Paolo 15, 00146 Rome, Italy; E-Mails: valentina.perri@opbg.net (V.P.); benedetta_russo6@msn.com (B.R.); 2 Division of Endocrinology, Children’s Hospital Bambino Gesù, Piazza S. Onofrio 4, 00165 Rome, Italy; E-Mails: antonino.crino@opbg.net (A.C.); riccardo.schiaffini@opbg.net (R.S.); marco.cappa@opbg.net (M.C.); 3 Research Laboratories, Children’s Hospital Bambino Gesù, Viale S. Paolo 15, 00146 Rome, Italy; E-Mail: ezio.giorda@opbg.net; 4 Consultant in Immunology, 00100 Rome, Italy; E-Mail: manuela.rosado@immunologyhomepage.com

**Keywords:** type 1 diabetes, T regulatory cells, programmed cell death 1-programmed cell death ligand 1

## Abstract

Type 1 diabetes is caused by autoreactive T cells that destroy pancreatic beta cells. Animal models suggested that a CD4^+^CD25^+^ population has a regulatory function capable of preventing activation and effector functions of autoreactive T cells. However, the role of CD4^+^CD25^high^ T cells in autoimmunity and their molecular mechanisms remain the subject of investigation. We therefore evaluated T regulatory cell frequencies and their PD-1 expression in the peripheral blood of long-standing diabetics under basal conditions and after CD3/CD28 stimulation. Under basal conditions, the percentages of T regulatory cells were significantly higher while that of T effector cells were significantly lower in patients than in controls. The ratio of regulatory to effector T cells was higher in patients than that in controls, suggesting that T regulatory cells were functional in patients. Percentages of total PD-1^+^, PD-1^low^ and PD-1^high^ expressing T regulatory cells did not change in patients and in controls. After stimulation, a defect in T regulatory cell proliferation was observed in diabetics and the percentages of total PD-1^+^, PD-1^low^ and PD-1^high^ expressing cells were lower in patients. Our data suggest a defective activation of T regulatory cells in long-standing diabetics due to a lower expression of PD-1 on their surface.

## 1. Introduction

Insulin-dependent diabetes mellitus (Type 1 diabetes, T1D) is an autoimmune disease in which pancreatic beta cells are destroyed by autoreactive T lymphocytes [1]. T1D is a multifactorial disorder; a close interaction of genetic and environmental factors underlies the disease pathogenesis. In the perinatal period, central tolerance mechanisms delete autoreactive clones within the thymus; peripheral autoreactive T cells escape and persist in the peripheral blood [2] suggesting that peripheral tolerance mechanisms also contribute to immune homeostasis. Early studies in animal models indicate that a CD4^+^CD25^+^ population has a regulatory function capable of preventing activation and effector functions of autoreactive T cells [3]. The onset and progression of the clinical disease appear to be the result of altered central tolerance deletion mechanisms within the thymus and halted function of regulatory T cells (Tregs). However, the role of CD4^+^CD25^high^ T cells in autoimmunity and, in particular, in the pathogenesis of T1D, remains the subject of intensive investigation [3] for the possible relevance of this subset in the development of innovative immunotherapeutic approaches.

To date, several studies have yielded conflicting results in determining the frequency and/or number of Tregs as well as their suppressive function in T1D patients. According to these studies, the number and frequency of peripheral Tregs were slightly high, significantly low or were normal in T1D patients [4]. Defective or normal suppressive function of Tregs were also reported [3,4,5,6,7,8,9,10,11,12,13,14,15,16,17,18,19,20,21,22,23,24,25]. It must be highlighted, however, that these discrepancies may derive from the fact that different studies utilized either peripheral blood lymphocytes or lymphocytes residing within lymph nodes, or were carried out on samples from new onset or long-standing diabetics of different age or ethnic origin.

The characterization of natural Tregs (nTregs) by cell surface markers identified them as CD25^high^ (interleukin-2 receptor α-chain) CD4^+^ T cells expressing low levels of CD127 (IL-7 receptor α-chain) [26]. FoxP3 (Forkhead box protein P3) is crucial for Treg function and is a master regulator of nTregs, a population inclusive of thymus-derived Tregs (tTregs) and peripheral-induced Tregs (pTregs) as well as *in vitro*-induced Tregs (iTregs). In addition to CD25, Treg cells express a variety of molecules engaged in Treg-mediated suppression, including: cytotoxic T lymphocyte antigen 4 (CTLA-4) [27]; CD39/CD73 [28]; LAG3 [29] and galectin-1 [30]; immunosuppressive cytokines (IL-10, TGF β, IL-35) [31]; and cytotoxic granzyme B [32]. Approximately 90% of CD4^+^CD25^+^CD127^low^ were found to express FoxP3 [33].

Several studies highlight the relevance of the programmed-cell death 1 (PD-1) also known as CD279 and programmed cell death ligand 1 (PD-L1) pathway in immunological tolerance and immune-mediated cell destruction [34]. This pathway is detected on different immunotypes: T, B lymphocytes, macrophages and some subtypes of dendritic cells (DCs) [34]. In particular, this pathway was detected on FoxP3 positive Treg cells, although its role in regulating their function and activation remains to be fully elucidated [34].

As a consequence of T cell stimulation, PD-1 directly affects the engagement of phosphatidylinositol 3-kinase (PI3K) activation mediated by CD28 [34,35,36,37] and is responsible for protein kinase B (Akt) activation. However, CTLA-4 acts, without affecting PI3K [37], by reducing Akt phosphorylation derived from protein phosphatase 2 (PP2A) activation [38,39].

The analyses *in vivo* of several animal models of disease, including the non-obese diabetic (NOD) mouse [40], have demonstrated the role of the PD-1/PD-L1 pathway in the development of autoimmunity [34,40,41,42,43,44,45,46,47].

PD-1 expression was detected more in CD4^+^ T cells than in CD8^+^ T cells, thus identifying a unique anergic subset that secretes IL-10 in RA synovial fluid [41]. PD-1 expression was also detected on lymphocytes; while PD-L1 expression was detected on epithelial cells from inflamed salivary glands of patients with Sjӧgren’s syndrome (SS) [42]. Single nucleotide polymorphisms (SNPs) in the PD-1 gene in humans were discovered and correlated with a higher risk of developing autoimmune diseases in certain ethnic groups [34,48,49]. Reduced basal and induced PD-1 expression was revealed on activated CD4^+^ T cells in SLE (systemic lupus erythematosus) patients homozygous for the PD1.3 polymorphism and reduced PD-1-mediated inhibition of early to intermediate stages of activation. In autologous mixed lymphocyte reactions (AMLR), a defective PD-1 induction on activated T cells of SLE patients was observed, especially among homozygotes [43]. In patients with lupus nephritis the PD-1/PD-L1 pathway was expressed at the renal tissue level, further suggesting its role in immunoregulation [43].

In autoimmune active generalized vitiligo (aGV), a deficiency in Treg frequency and a decreased expression of Treg-associated parameters (TGFβ-CCL21) were observed [44,45] together with an increased percentage of PD-1^+^ Tregs, thus implying a role of PD-1/PD-L1 pathway in Treg exhaustion [45].

As regards T1D, a decreased expression of *PD-1* gene was observed in CD4^+^ T cells of patients with autoimmune T1D and in recent studies [46,47], CD4^+^ T cells of Japanese T1D patients carrying the 7785 C/C genotype of the *PDCD1* gene showed lower PD-1 expression than those with the C/T and T/T genotypes. These results indicate that the lower PD-1 expression might contribute to the development and/or maintenance of T1D through T cell activation.

In the light of the foregoing discussion and to further elucidate the putative role of PD-1 in T cell function in autoimmunity development, we examined PD-1 expression in activated CD4^+^CD25^+^ T cells and Treg population after CD3/CD28 stimulation of peripheral blood lymphocytes of a group of T1D patients and of a group of healthy controls.

## 2. Results and Discussion

### 2.1. Study Population

The study population included 10 T1D patients and 10 healthy controls. All were patients with long-standing disease. The mean actual age of T1D patients was 18.4 years (ranging from 12 to 27 years; 3 males, 7 females). The mean age at disease onset was 4.6 years (ranging from one to 10 years) and the mean duration of the disease was 13.8 years (ranging from 10 to 17 years). The mean age of the controls (healthy donors, HD) was 23 years (ranging from 18 to 30 years). Demographic and clinical characteristics of patients are shown in Table 1.

In addition to T1D (Table 1), seven patients developed autoimmune thyroid disease (AT), six patients developed Hashimoto’s thyroiditis (autoimmune polyglandular syndrome Type 3 variant, APS3v), which was confirmed by the presence of circulating thyroglobulin (Tg) and thyroperoxidase (TPO) autoantibody specificities (Abs) and echographic pattern of diffuse hypoechogenicity, and one patient had Basedow’s disease. Two patients with APS3v also presented autoimmune gastritis (AG), which was confirmed by the presence of parietal cell (PCA) Abs. In addition to T1D, one patient had celiac disease (CD) (confirmed by the presence of transglutaminase (tTGA) Abs at diagnosis) and vitiligo. Two patients with T1D and Hashimoto’s thyroiditis also had vitiligo.

**Table 1:** Demographic, clinical, laboratory and metabolic characteristics of the long-standing T1D patients recruited for the study.

Pt	Sex	Age of Disease Onset	Actual Age	Duration of Disease	Associated Diseases	Islet-Related Abs	Other Abs	HbA1c
1	M	3	16	13		GADA: 0.1; IA2: 0.1	TPO: <28.0; Tg: <20; tTGA: 0.2	**69**
2	F	10	27	17	AT	GADA: 0.4; IA2: **4.6**	TPO: >**1300**; Tg:	**72**
3	F	6	22	16	AT; AG	GADA: **17.0**; IA2: 1.0	TPO: >**1300**; Tg: 27.7; tTGA: 0.2; PCA pos	**73**
4	F	4	16	12	AT; vitiligo	GADA: 0.1; IA2: **2.7**	TPO: <28.0; Tg: <20.0; tTGA: 0.2	**85**
5	F	2	12	10	AT	GADA: 0.3; IA2: **1.5**	TPO: **266.7**; Tg: <20.0; tTGA: 0.2	**78**
6	F	3	20	17		GADA: **1.6**; IA2: **1.6**	TPO: <28.0; Tg: <20.0; tTGA: 0.2	**72**
7	M	1	13	12	AT	GADA: **1.3**; IA2: 0.1	TPO: **498.9**; Tg: <20.0; tTGA: 0.3	**62**
8	M	3	19	16	Basedow	GADA: 0.9; IA2: 0.4	TPO: <28.0; Tg: 24.0; tTGA: 0.2	**64**
9	F	10	24	14	AT; AG; vitiligo	GADA: **120.0**; IA2: **2.6**	TPO: >**1300**; Tg: **397.0**; tTGA: 0.9; PCA pos	**93**
10	F	4	15	11	CD; vitiligo	GADA: **2.3**; IA2: 0.1	TPO: <28.0; Tg: <20.0; tTGA: 0.5	**70**

PCA pos = positive for PCA Abs; Islet-related Abs reference values: glutamic acid decarboxylase isoform 65 (GADA) < 1 Units/milliliter (U/mL); protein tyrosine phosphatase insulinoma-associated antigen 2 (IA2) < 1.1 U/mL; other Abs reference values: TPO < 60 U/mL; Tg < 40 U/mL; tTGA < 4 U/mL. tTGA tested during long-standing disease and under diet restriction. HbA1c reference value: >48 millimoles/mole (mmol/mol). Pathological values are indicated in bold. Pt = patient.

### 2.2. Analysis of T Regulatory and T Effector Cell Subsets after Four and Six Days of Culture under Standard Basal Conditions

**Figure 1:**
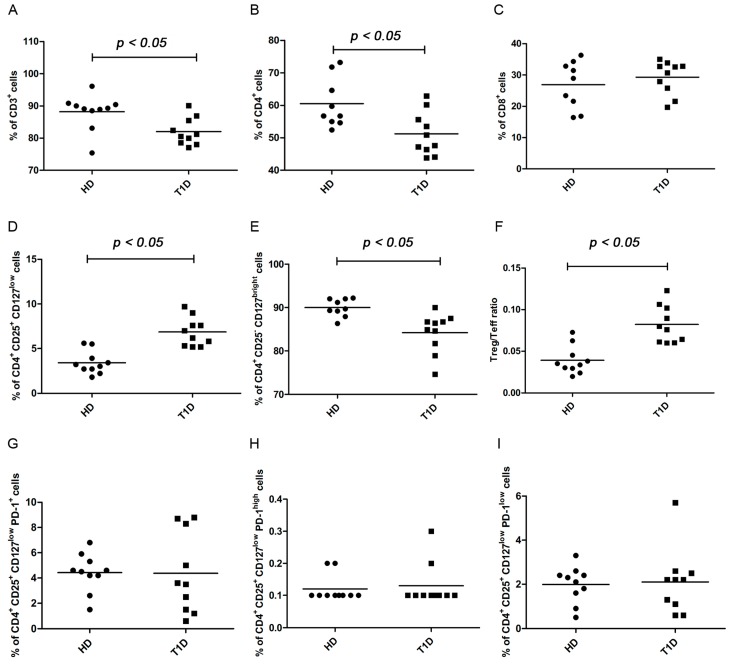
T cell phenotype and percentages of PD-1^+^ Tregs in healthy controls and in T1D patients after four days of culture under standard basal conditions. Previously frozen PBMC samples from healthy control donors and from T1D patients were stained with antibodies to CD3, CD4, CD25 and CD127 and analyzed by Flow cytometric analysis (FACS) to determine the relative frequency of CD3^+^ (**A**); CD4^+^ (**B**); CD8^+^ (**C**); Treg (**D**); Teff (**E**) cells and the ratio of Treg and Teff percentages (**F**); Graphs (**G**–**I**) show the frequency after four days of culture of total PD-1^+^, PD-1^high^ and PD-1^low^ Treg subfractions respectively in healthy controls and T1D patients. In all graphs, horizontal lines represent the mean frequency and each symbol represents an individual: black circle represents the normal and square dot the diabetic. Frequencies refer to analyzed events within flow-cytometry gates as shown in representative dot plots in Figure S2.

After four days of culture in RPMI supplemented with interleukin-2 (IL-2) under standard basal conditions, the percentages of total CD3^+^ T cells were significantly lower in T1D patients than in controls (Figure 1A, Figure S1A, Kolmogorov-Smirnov test *p* < 0.05; Mann Whitney test *p* = 0.0147). Percentages of CD4^+^ T cells were higher in HD than in T1D peripheral blood mononuclear cells (PBMC) (Figure 1B, Figure S1B, Kolmogorov-Smirnov test *p* > 0.10; unpaired *t* test with Welch’s correction *p* = 0.0124) and CD8^+^ T cells had not changed significantly in T1D patients as compared to controls (Figure 1C, Figure S1C, Kolmogorov-Smirnov test *p* > 0.10; unpaired *t* test with Welch’s correction *p* = 0.4425).

In CD4^+^ T cells, CD4^+^/CD25^+^/CD127^low^ Tregs were significantly higher in T1D patients than in controls (Figure 1D, Figure S1D, Kolmogorov-Smirnov test *p* > 0.10; unpaired *t* test with Welch’s correction *p* < 0.0001) (Figure S2C,G), while CD4^+^/CD25^-^/CD127^high^ T effector cells (Teffs) were significantly lower (Figure 1E, Figure S1E, Kolmogorov-Smirnov test *p* > 0.10; unpaired *t* test with Welch’s correction *p* = 0.0036). Further, the Treg/Teff ratio was significantly higher in T1D patients than in controls (Figure 1F, Figure S1F, Kolmogorov-Smirnov test *p* > 0.10; unpaired *t* test with Welch’s correction *p* = 0.0002), suggesting that Tregs are functional in T1D patients. Similar results were obtained after six days of culture under standard basal conditions for the percentages of CD3^+^ (Figure S3A, Kolmogorov-Smirnov test *p* > 0.10; unpaired *t* test with Welch’s correction *p* = 0.0401), while the difference for total CD4^+^ T cells did not reach statistical significance being equally low in both groups, which probably reflects apoptosis (Figure S3B, Kolmogorov-Smirnov test *p* > 0.10; unpaired *t* test with Welch’s correction *p* = 0.1955). After six days, CD8^+^ T cells (Figure S3C, Kolmogorov-Smirnov test *p* > 0.10; unpaired *t* test with Welch’s correction *p* = 0.9824), Treg cells (Figure S3D, Kolmogorov-Smirnov test *p* > 0.10; unpaired *t* test with Welch’s correction *p* < 0.0001), Teff cells (Figure S3E, Kolmogorov-Smirnov test *p* < 0.05; Mann Whitney test *p* = 0.0025), and Treg/Teff ratio (Figure S3F, Kolmogorov-Smirnov test *p* > 0.10; unpaired *t* test with Welch’s correction *p* = 0.0002), showed similar results to those obtained after four days. No correlation was observed between immune cell subsets and HbA1c values (Table S1) in agreement with the results of previous studies in established T1D patients [10,11].

#### PD-1 Expression

Regulatory T cells were identified as CD4^+^CD25^+^CD127^low^ and PD-1 expression as indicated in Figure S2C. Percentages of PD-1 positive cells (Figure 1G, Figure S1G, Kolmogorov-Smirnov test *p* > 0.10; unpaired *t* test with Welch’s correction *p* = 0.9650) (Figure S2D,H) within the Treg population did not change significantly among healthy controls and T1D patients after four days under standard basal conditions; there was also no difference in the percentage of PD-1^high^ (Figure 1H, Figure S1H, Kolmogorov-Smirnov test *p* < 0.05; Mann Whitney test *p* = 0.9568) and PD-1^low^ subfractions (Figure 1I, Figure S1I, Kolmogorov-Smirnov test *p* < 0.05; Mann Whitney test *p* = 0.8797). Similar results were obtained after six days of culture under standard basal conditions for PD-1 positive (Figure S3G, Kolmogorov-Smirnov test *p* > 0.10; unpaired *t* test with Welch’s correction *p* = 0.9001), PD-1^high^ (Figure S3H, Kolmogorov-Smirnov test *p* < 0.05; Mann Whitney test *p* = 0.1234) and PD-1^low^ expressing cells (Figure S3I, Kolmogorov-Smirnov test *p* > 0.10; unpaired *t* test with Welch’s correction *p* = 0.6142).

### 2.3. Analysis of T Regulatory and T Effector Cell Subsets after Four and Six Days of CD3/CD28 Costimulation

**Figure 2:**
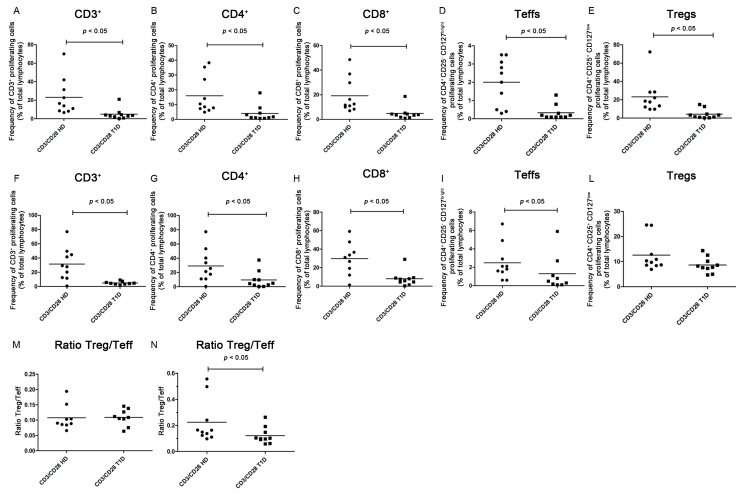
T cell, Teff, Treg proliferative responses and the Treg/Teff ratio after CD3/CD28 stimulation in healthy controls and in T1D patients. 5-chloromethylfluorescein diacetate (CMFDA)-labeled PBMC from healthy controls and from T1D patients were stimulated with CD3/CD28-coated beads for four days (upper panels, A–E and M) and six days (bottom panels, F–L and N). Graphs show the frequency of CD3^+^, CD4^+^, CD8^+^, Teff, Treg proliferating cells after 4 (**A**–**E**) and 6 days (**F**–**I**,**L**) and Treg/Teff ratio after four days (**M**) and six days (**N**) of culture in healthy controls and T1D patients. Proliferation was calculated as percentages of CMFDA-low cells within the total subset after CD3/CD28 stimulation over the percentages of CMFDA-low in RPMI unstimulated cultures. In all graphs, horizontal lines represent the mean frequency and each symbol represents an individual: black circle represents the normal and square dot the diabetic.

Anti-CD3/CD28 costimulation for four days significantly induced T cell proliferation in both healthy controls (paired *t* test *p* = 0.0069) and in T1D patients (paired *t* test *p* = 0.0437) (Figure S4A–H, data not shown). The number of proliferating CD3^+^ cells was higher in PBMC cultures obtained from the healthy controls than those from the patients (Figure 2A, Figure S5A, Kolmogorov-Smirnov test *p* < 0.05; Mann Whitney test *p* = 0.0005).

Anti-CD3/CD28 costimulation induced proliferation of CD4^+^ T cells in both T1D patients (paired *t* test *p* = 0.0447) and healthy controls (paired *t* test *p* = 0.0039) (Figure S6A,B) and of CD8^+^ T cells in both healthy controls (paired *t* test *p* = 0.0037) and T1D patients (paired *t* test *p* = 0.0362) (Figure S6C,D). However, the proliferative response of CD4^+^ T cells was significantly higher in healthy controls than in T1D patients (Figure 2B, Figure S5B, Kolmogorov-Smirnov test *p* < 0.05; Mann Whitney test *p* = 0.0032). The number of proliferating CD8^+^ T cells was higher in healthy controls than in T1D patients (Figure 2C, Figure S5C, Kolmogorov-Smirnov test *p* < 0.05; Mann Whitney test *p* = 0.0005). After six days of stimulation, we observed the same results for CD3^+^ cells in healthy controls (paired *t* test *p* = 0.0019) and T1D patients (paired *t* test *p* < 0.0001) (data not shown), for CD4^+^ (paired *t* test *p* = 0.0034 for healthy controls and *p* = 0.0421 for T1D patients) (Figure S6E,F) and for CD8^+^ T cells (paired *t* test *p* = 0.0014 for healthy controls and *p* = 0.0443 for T1D patients) (Figure S6G,H). Similarly, the number of proliferating CD3^+^ cells was higher in PBMC obtained from the healthy controls than from the patients (Figure 2F, Figure S5F, Kolmogorov-Smirnov test *p* > 0.10; unpaired *t* test with Welch’s correction *p* = 0.048). The proliferative response of CD4^+^ T cells was significantly higher in healthy controls than in T1D patients (Figure 2G, Figure S5G, Kolmogorov-Smirnov test *p* < 0.05; Mann Whitney test *p* = 0.0209). The number of proliferating CD8^+^ T cells was higher in healthy controls than in T1D patients (Figure 2H, Figure S5H, Kolmogorov-Smirnov test *p* < 0.05; Mann Whitney test *p* = 0.0057).

After four days of stimulation, Teffs proliferated significantly in healthy controls (paired *t* test *p* = 0.0182) but not in T1D patients (paired *t* test *p* = 0.2091) (Figure S7A,B). The same results were obtained after six days of stimulation for healthy controls (paired *t* test *p* = 0.0107) and T1D patients (paired *t* test *p* = 0.0855) (Figure S7E,F). The percentage of proliferating Teffs was higher in the healthy controls than in the patients, both, after four days (Figure 2D, Figure S5D, Kolmogorov-Smirnov test *p* < 0.05; Mann Whitney test *p* = 0.0011) and after six days of stimulation (Figure 2I, Figure S5I, Kolmogorov-Smirnov test *p* < 0.05; Mann Whitney test *p* = 0.0453).

Tregs proliferated significantly after four days in the PBMC of both healthy controls (paired *t* test *p* = 0.0061) and in T1D patients (paired *t* test *p* = 0.0420) (Figure S7C,D). Similar results were obtained after six days in PBMC of healthy controls (paired *t* test *p* = 0.0028) and T1D patients (paired *t* test *p* = 0.0150) (Figure S7G,H). However, the percentage of proliferating Tregs was significantly higher in healthy controls than in patients after four days (Kolmogorov-Smirnov test *p* < 0.05; Mann Whitney test *p* = 0.0005) (Figure 2E, Figure S5E), while no significant difference was observed after six days (Kolmogorov-Smirnov test *p* < 0.05; Mann Whitney test *p* = 0.0820) (Figure 2L, Figure S5L).

After four days of stimulation, the Treg/Teff ratio increased in both, healthy controls (paired *t* test *p* = 0.0001) and T1D patients (paired *t* test *p* = 0.0010) (Figure S8A,B) but no significant difference was observed among the healthy controls *versus* the patients (Kolmogorov-Smirnov test *p* < 0.05; Mann Whitney test *p* = 0.4894) (Figure 2M, Figure S5M). After six days of stimulation also Treg/Teff increased in both healthy controls (paired *t* test *p* = 0.0077) and T1D patients (paired *t* test *p* = 0.0247) (Figure S8C,D). However, the Treg/Teff ratio increased significantly in healthy controls *versus* T1D patients after six days (Kolmogorov-Smirnov test *p* < 0.05; Mann Whitney test *p* = 0.0355) (Figure 2N, Figure S5N), suggesting that, in T1D patients, Tregs exert an inhibitory effect on Teffs although less efficiently than in controls.

#### PD-1 Expression

**Figure 3:**
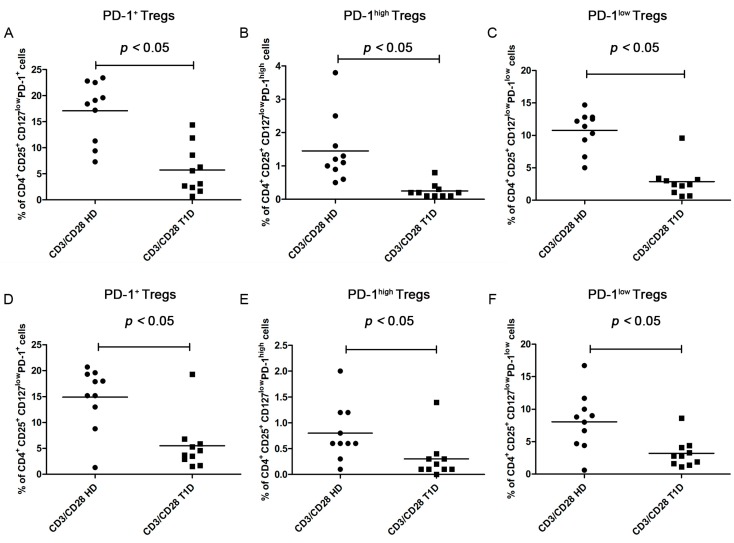
Percentages of PD-1^+^ Tregs after CD3/CD28 stimulation in healthy controls and in T1D patients. The graphs show the frequency of total PD-1^+^, PD-1^high^, and PD-1^low^ subfractions after four days (**A**–**C**) and six days (**D**–**F**) of stimulation.

After four days of CD3/CD28 stimulation, PD-1 positive cells significantly increased in healthy controls (paired *t* test *p* < 0.0001) but did not change significantly in T1D patients (paired *t* test *p* = 0.0501) (Figure S9A,B). The same results were obtained for PD-1^high^ expressing cells in healthy controls (paired *t* test *p* = 0.0025) and T1D patients (paired *t* test *p* = 0.0963) (Figure S9C,D) and for the PD-1^low^ subfraction (in HD paired *t* test *p* < 0.0001; in T1D patients paired *t* test *p* = 0.0801) (Figure S9E,F). The percentage of total PD-1^+^ (Figure 3A, Kolmogorov-Smirnov test *p* > 0.10; unpaired *t* test with Welch’s correction *p* = 0.0001), PD-1^high^ (Figure 3B, Kolmogorov-Smirnov test *p* < 0.05; Mann Whitney test *p* < 0.0001) and PD-1^low^ (Figure 3C, Kolmogorov-Smirnov test *p* < 0.05; Mann Whitney test *p* = 0.0004) expressing Tregs was higher after stimulation in healthy controls than in T1D patients.

After six days of CD3/CD28 stimulation, PD-1 positive cells significantly increased in healthy controls (paired *t* test *p* = 0.0001), but did not change significantly in T1D patients (paired *t* test *p* = 0.7521) (Figure S9G,H). The same results were obtained for PD-1^high^ expressing cells in healthy controls (paired *t* test *p* = 0.0049) and in T1D patients (paired *t* test *p* = 0.6208) (Figure S9I,L) and for the PD-1^low^ subfraction (in HD paired *t* test *p* = 0.0011; in T1D patients paired *t* test *p* = 0.1698) (Figure S9M,N).

After six days of stimulation, total PD-1^+^ Tregs were significantly higher in healthy controls than in T1D patients (Figure 3D, Kolmogorov-Smirnov test *p* < 0.05; Mann Whitney test *p* = 0.0125). The same results were obtained for PD-1^high^ (Figure 3E, Kolmogorov-Smirnov test *p* < 0.05; Mann Whitney test *p* = 0.0128) and PD-1^low^ expressing Tregs (Figure 3F Kolmogorov-Smirnov test *p* > 0.10; unpaired *t* test with Welch’s correction *p* = 0.0082). These data indicate a less efficient response in the patient group.

**Figure 4:**
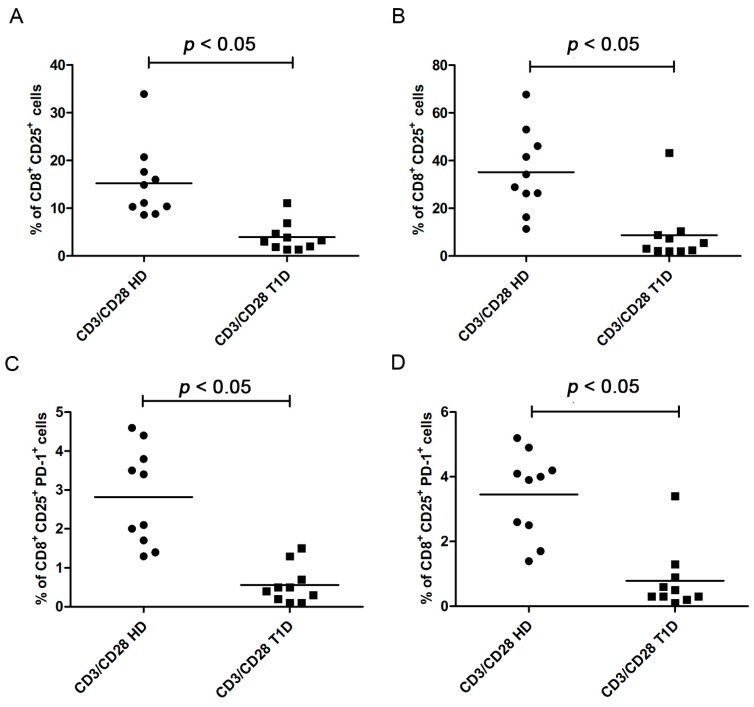
Analysis of CD8^+^CD25^+^ subsets after CD3/CD28 stimulation in healthy controls and in T1D patients. **Upper** panel graphs show the percentages of CD8^+^CD25^+^ cells after four days (**A**) and six days (**B**) of stimulation in healthy controls and in T1D patients; **Bottom** panel graphs show the percentages of CD8^+^CD25^+^ PD-1^+^ cells after four days (**C**) and six days (**D**).

When analyzing the proliferating Tregs, we found a significantly lower PD-1 expression in T1D patients than in healthy controls, both, after four days (total PD-1^+^: Kolmogorov-Smirnov test *p* < 0.05, Mann Whitney test *p* = 0.0065; PD-1^high^: Kolmogorov-Smirnov test *p* < 0.05, Mann Whitney test *p* = 0.0164; PD-1^low^: Kolmogorov-Smirnov test *p* < 0.05, Mann Whitney test *p* = 0.0166, data not shown) and six days of stimulation (total PD-1: Kolmogorov-Smirnov test *p* < 0.05, Mann Whitney test *p* = 0.0412; PD-1^high^: Kolmogorov-Smirnov test *p* < 0.05, Mann Whitney test *p* = 0.0464; PD-1^low^: Kolmogorov-Smirnov test *p* < 0.05, Mann Whitney test *p* = 0.0355, data not shown).

The analysis of the CD8^+^ subsets, that proliferate less vigorously in T1D patients than in controls (see above), revealed that the percentage of CD8^+^CD25^+^ cells was significantly lower in T1D patients than in controls, both, after four days (Figure 4A, Kolmogorov-Smirnov test *p* > 0.10; unpaired *t* test with Welch’s correction *p* = 0.0013) and after six days of stimulation (Figure 4B, Kolmogorov-Smirnov test *p* < 0.05; Mann Whitney test *p* = 0.0005). Percentages of PD-1^+^ cells in the CD8^+^CD25^+^ subset were lower in T1D patients than in controls, both, after four days (Figure 4C, Kolmogorov-Smirnov test *p* > 0.10; unpaired *t* test with Welch’s correction *p* = 0.0002) and six days of stimulation (Figure 4D, Kolmogorov-Smirnov test *p* < 0.05; Mann Whitney test *p* = 0.0001). These data indicate that CD8^+^ T cells present a lower level of activation in T1D patients.

**Figure 5:**
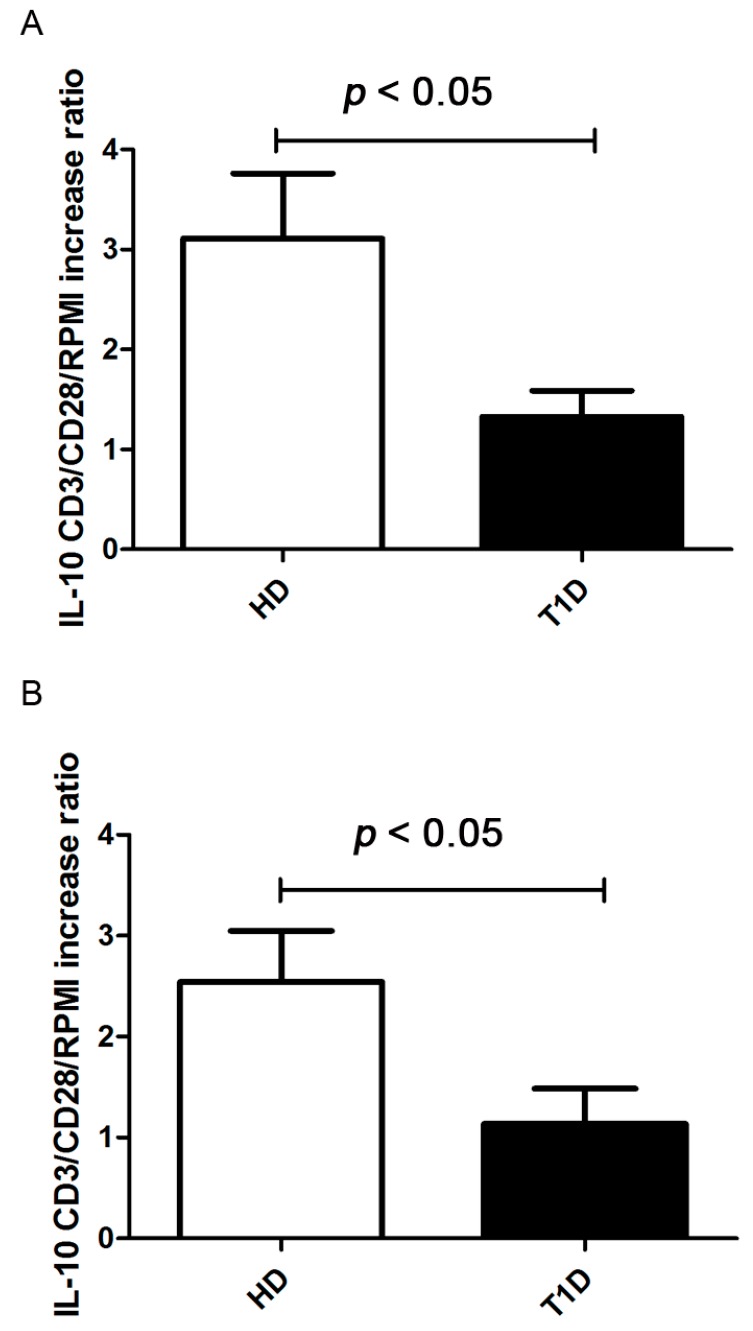
IL-10 secretion after four and six days of CD3/CD28 stimulation in PBMC of T1D patients compared to healthy controls. Ratio of increase of IL-10 concentrations (expressed as picograms/milliliter (pg/mL) was calculated in CD3/CD28-stimulated over unstimulated supernatants. Graphs show a significant reduction of IL-10 secretion in T1D patients compared to healthy controls, both, after four days (**A**) (Kolmogorov-Smirnov test *p* > 0.10; unpaired *t* test with Welch’s correction *p* = 0.0275) and six days (**B**) (Kolmogorov-Smirnov test *p* < 0.05; Mann Whitney test *p* = 0.0147) of CD3/CD28 stimulation.

### 2.4. Treg Immunosuppressive Function

In order to address regulatory T cell function in our patients, we measured the ability of Tregs to secrete interleukin-10 (IL-10) upon stimulation. Regulatory cytokine IL-10 levels were significantly higher in supernatants of PBMC cultures of healthy controls than in T1D patients, both, after four and six days of CD3/CD28 stimulation (Figure 5). This supports the hypothesis that Treg cells from T1D patients with stable, long-standing disease have a reduced suppressive function.

### 2.5. Discussion

In recent years, the complexity of Tregs and their effects on Teff function in T1D patients has been the subject of intensive investigation aimed at exploiting their use in novel cell-based therapies to restore self-tolerance. Nevertheless, contrasting data were reported in the literature regarding Treg frequencies and suppressive function (see above). Putnam *et al*. [50] already demonstrated that circulating Tregs can be expanded in the peripheral blood of patients at onset of T1D and are able to reach significant concentrations to be used for autologous transplantation. Another avenue of application is their theoretical utility in preventing rejection of allogeneic islet grafts.

This innovative field requires further knowledge on the properties of this T cell subset and on its genetic control as well as on its phenotype through the analysis of functional molecules in comparison with healthy controls. Few initial studies pointed to the influence of the PD-1/PD-L1 regulatory pathway on Tregs in diabetic patients [34,46,47,48,49]. Several investigations have highlighted that this pathway can be introduced as a prime opportunity to personalised medicine through the development of regulatory PD-1 agonists [34].

In the light of the contrasting data regarding Treg frequencies and suppressive function in different groups of diabetics, our original approach was to assess their functionality by analysing the expression of the PD-1/PD-L1 regulatory pathway in a homogeneous group of patients recruited among those having disease of a certain duration from 11 to 17 years in comparison with healthy controls.

Few initial studies so far pointed to the influence of the PD-1/PD-L1 regulatory pathway on Tregs in diabetic patients [34,46,47,48,49] without demonstrating a direct correlation with the activation of this cell subset. As regards the PD-1/PD-L1 pathway, PD-1 signaling is required for the maintenance of functional regulatory CD4^+^CD25^+^FoxP3^+^ T cells that can control autoimmunity as shown by Wong *et al*. [51] in the F1 lupus-like disease mouse model. Raimondi *et al*. [52] observed that 90% of naturally arising FoxP3^+^ Tregs did not express the inhibitory receptor PD-1 on their surface, but retained PD-1 intracellularly. A similar phenomenon was observed on CD4^+^CD25^high^ T cells isolated from the PBMC of healthy donors, whereas activated T cells expressed high levels of surface PD-1 that paralleled up-regulation of CD25 during effector cell expansion. Tregs translocate PD-1 to the cell surface when stimulated via the T cell receptor (TCR) [52]. PD-1 expression is inversely correlated with FoxP3 expression in CD4^+^ Tregs and the expression of low levels of PD-1 on CD4^+^ Tregs promotes their regulatory capacity. PD-1^low^ CD4^+^ Tregs (compared to PD-1^high^ CD4^+^ Tregs) had increased transforming growth factor beta (TGF-β) production and were resistant to apoptosis. However, very low levels of PD-1 expression resulted in a loss of the regulatory capacity of CD4^+^ Tregs. Thus, PD-1 expression modulates the suppressive function of CD4^+^ Tregs in a quantitative manner and an effective function of CD4^+^ Tregs depends on a low, but not absent, expression of PD-1 [51].

Regarding the functional role of the PD-1/PD-L1 pathway, Franceschini *et al.* [53] suggested that blocking PD-L1 *in vitro* leads to the expansion of Tregs by controlling STAT-5 (signal transducer and activator of transcription 5) phosphorylation. This implies that Treg function can be contra-regulated by PD-1/PD-L1 engagement. Gotot *et al.* [54] reported that Tregs suppress autoreactive B cells via PD-L1 and such suppression requires expression of PD-1 on autoreactive B cells and of two PD-1 ligands on Treg cells.

Previous studies have shown that PD-1 deficiency or administration of a monoclonal antibody to PD-1 in NOD mice, results in accelerated and exacerbated diabetes [40,55]. Compared with wild-type NOD mice, an enhanced islet infiltration of CD4^+^ and CD8^+^ T lymphocytes was found. In another study [56], PD-1 deficiency in antigen-specific CD4^+^ T cells of NOD mice accelerated T1D development and increased islet T cell infiltrates and T cell numbers in spleen and pancreatic lymphnodes. The initial study of Tsutsumi *et al.* [46], observed a decreased PD-1 expression in CD4^+^ T lymphocytes of a small and heterogeneous group of T1D patients as compared to healthy controls, suggesting that PD-1 plays a role in the development and maintenance of the disease. In a recent report by Fujisawa *et al.* [47], a lower PD-1 expression in CD4^+^ T cells contributed to the development of T1D through T cell activation. Significantly, a lower PD-1 expression was found in patients with the 7785 C/C genotype. There was no significant correlation between the disease duration and the frequency of PD-1 expression. Thus, PD-1 expression may be used as a parameter to address regulatory T cell function.

In our protocol, PBMC obtained from patients after four to six days of CD3/CD28 stimulation were examined. A first analysis showed that after four days of culture under standard basal conditions, the percentages of CD3^+^ and CD4^+^ T cells were lower in T1D patients than in healthy controls, while no difference was observed for CD8^+^ T cells. After six days, there was also no difference for CD4^+^ T cells due to the possible influence of apoptotic events (see above) in early activated cells of healthy controls. After stimulation for four and six days, the number of proliferating CD3^+^, CD4^+^ and CD8^+^ T cells was lower in T1D patients than in controls. In this regard, in a previous study, a constitutive impaired TCR/CD3-mediated activation of T cells in diabetics was observed [57]. This defect was linked neither to the class II major histocompatibility complex (MHC) genotype, metabolic disturbances nor to the presence of specific autoantibodies. The inefficient activation of T cells was not related to a lower capacity of CD28 to transduce co-stimulatory signals because under CD3/CD28 stimulation, proliferative responses were similar between patients and controls and the PBMC from patients had a higher reactivity to increasing concentrations of IL-2 [57].

Under standard basal conditions, the percentages of Tregs were significantly higher, while Teffs were significantly lower in T1D patients than in controls. This may be the result of the long-standing disease, which would explain the discrepancy with previous studies that reported a lower frequency of Tregs at disease onset. Further, Treg/Teff ratio was higher in T1D patients than in healthy controls, suggesting that Tregs were functional in patients. Percentages of total PD-1^+^, PD-1^low^ and PD-1^high^ positive cells did not change among patients and controls within the Treg compartment.

As regards T1D patients, either at disease onset or in the long-standing disease, the percentages of both CD4^+^CD25^+^ and CD4^+^CD25^high^ T cells were found not statistically different than those from healthy controls [3,4,6,9,13,58,59] in contrast to other studies where T1D patients exhibited reduced mean percentages of resting immunoregulatory CD4^+^CD25^+^ T cells [5,24,58,60,61]. In keeping with our findings, two previous studies also found that activated CD45RA-FoxP3^high^ Tregs were found higher in the peripheral blood of diabetics due to compensatory mechanisms to their decreased suppressive activity [18,22].

In our study, after four days of CD3/CD28 stimulation, Tregs proliferate in both, PBMC from healthy controls and that from diabetics. However, the percentage of proliferating Tregs was higher in healthy controls than in T1D patients. In parallel, Teffs proliferate significantly more in healthy controls than in patients at both time intervals. The Treg/Teff ratio did not change significantly in the two groups after four days but the ratio was higher after six days in healthy controls, which indicates that in T1D, Tregs exert an inhibitory effect, albeit, less efficient than in healthy controls. In addition, the Treg/Teff ratio was lower in healthy controls than in T1D patients before stimulation, further supporting the hypothesis that Tregs are functionally defective in diabetic patients. This was further confirmed by the presence of lower concentrations of IL-10 detected in culture supernatants of PBMC from T1D patients than in healthy controls, both, after four and six days of stimulation. Furthermore, due to a defective activation of Tregs, percentages of total PD-1 positive, PD-1^low^ and PD-1^high^ were significantly higher in healthy controls than in T1D patients.

In support of our findings, functional studies have previously demonstrated a defective *in vitro* suppressive activity of Tregs in T1D patients [5,6,9,13,24,58,62] and in autoimmune polyglandular syndrome Type 2 (APS-II) patients with long-standing T1D [63]. However, some studies report a similar suppressive function in long-standing diabetics than in healthy controls [3,58].

In addition, we observed that, within the CD8+/CD25+ population, which proliferates less vigorously in T1D patients, the percentage of total PD-1 positive cells was also significantly reduced in T1D patients, suggesting a defect in the ability to up-regulate PD-1 and thus use the PD-1/PD-L1 pathway.

## 3. Materials and Methods

### 3.1. Subjects

The patient group consisted of 10 long-standing T1D patients who were recruited at the Department of Endocrinology at Bambino Gesù Children’s Hospital (OPBG) over the past two years.

Patients’ sera were tested for diabetes-related Abs *i.e.*,: GADA; IA2 and insulin (IAA) Abs by radioimmunoassay (RIA); Tg; TPO and tTGA Abs by chemiluminescence (ADVIA Centaur analyzer: Siemens Healthcare, Germany) and PCA, the adrenal cortex (ACA) and islet cell Abs by indirect immunofluorescence (IFL). Mean HbA1c values of patients were 73.8 ± 9.4 mmol/mol (cutoff value 48 mmol/mol), indicating a poor metabolic control, which required insulin therapy adjustments. The control group consisted of 10 HD recruited from the Blood Transfusion Centre at the OPBG; they had no history of autoimmunity, and no circulating Abs.

All control subjects were matched for sex, age, ethnic origin and geographical area. All enrolled patients and controls were unrelated. All subjects were recruited in the investigation after obtaining written informed consent. The study was approved by the local Institutional Review Board (IRB) of the OPBG, which regulates the use of human samples for experimental studies. The written informed consent for the children was obtained from the next of kin. The participants’ consent was recorded using a paper-based inventory system. The IRB approved the consent procedure (170 CM/lb, 4 May 2011).

### 3.2. Cell Preparation

PBMC were separated by Ficoll-Hypaque (Histopaque, Sigma-Aldrich Chemical: St Louis, MO, USA) from sodium heparinized venous blood samples (5–10 mL) according to standard protocols [64].

### 3.3. Stimulation of PBMC with CD3/CD28-Coated Beads and Proliferation Assay

Before stimulation, PBMC were labeled with CMFDA (CellTracker, Invitrogen, Molecular Probes: Eugene, OR, USA) at a final concentration of 0.1 μg/mL and cultured at 7.5 × 10^5^ cells per well in 96-well flat-bottomed plates (Falcon, Labware BD Biosciences: Oxnard, CA, USA) in complete RPMI 1640 medium (GIBCO/BRL, Invitrogen: Gaithersburg, CA, USA) supplemented with 10% fetal calf serum (FBS, Hyclone: South Logan, UT, USA), l-glutamine (2 mM) and 1% penicillin/streptomycin according to established protocols [64]. The cells were stimulated with Dynabeads^®^ Human T-activator CD3/CD28 (Life Technologies AS, Oslo, Norway) at a bead-to-cell ratio of 1:50. We used this suboptimal anti-CD3/CD8 bead-to-cell ratio because preliminary tests demonstrated that it was supposed to mimic a real physiological situation and more appropriate for detecting subtle immunomodulatory activities than the recommended 1:1 ratio known to generate a maximal proliferative response [65]. In addition, cultures were supplemented with IL-2 (5 International Units (IU)/mL, Sigma Aldrich: St. Louis, MO, USA). IL-2 was added to the cultures because we found that a low dose improved cell survival in cryopreserved pathological samples without altering cell function and affecting the PD-1/PD-L1 inhibitory pathway [66]. The cells were incubated for four and six days at 37 °C in a humidified atmosphere containing 5% CO_2_. Cell proliferation was assessed on days four and six by flow cytometry using a FACSCanto II analyzer (Becton and Dickinson (BD): Sunnyvale, CA, USA) and PC FACSDiva software (BD Biosciences: San Jose, CA, USA). Fifty thousand events per sample were analyzed [64].

### 3.4. FACS

After four- and six-days of CD3/CD28 beads stimulation, PBMC were harvested from the culture plates, separated from the beads using a Dynal MPC^®^-6 magnetic particle concentrator (Dynal Biotech ASA: Oslo, Norway) and washed by centrifugation at 1200 rpm for five minutes at room temperature (RT) in wash buffer (2% FBS in phosphate buffered saline (PBS)). To identify T cell subsets, single-cell suspensions were stained with the following directly conjugated monoclonal antibodies (MoAb): CD3-Alexa Fluor 700 (1:40 dilution; BD Biosciences: San Diego, CA, USA); CD4-PerCP-Cy5.5; CD25-phycoerithrin (PE); CD127-Alexa Fluor 647 (1:5 dilution; Human regulatory T-cell sorting kit, BD Biosciences) and CD279 (PD-1)-PE-Cy7 (1:50 dilution; eBioscence, Inc.: San Diego, CA, USA). The cells were incubated for 20 min in the dark at 4 °C. After labeling, the cells were washed by centrifugation at 1200 rpm for 5 min at 4 °C in wash buffer. The CD4^+^/CD25^-^/CD127^high^ and CD4^+^/CD25^+^/CD127^low^ cells were identified Teffs and Tregs, respectively. The identity of Tregs was confirmed by the expression of FoxP3 [33,65,67]. For the analysis of FoxP3 positive cells, cells were fixed and permeabilized using FoxP3 buffer salt kit (BD Cytofix/Cytoperm 51-2090KZ) according to the manufacturer’s guidelines. Single cell suspensions were incubated in the dark for 30 min at RT with fluorescein (FITC)-conjugated MoAb (clone 259D/C7) directed against human FoxP3.

Data were acquired on the FACSCanto II (BD). Flow cytometry profiles were analyzed using the FACSDiva software (BD Biosciences). Dead cells were excluded from the analysis by side/forward scatter gating [64]. A minimum of fifty thousand events, gated on living cells, were collected per data set. In order to exclude that recently activated T cells expressing CD25 would be wrongly categorized as Tregs, a tight lymphocyte gate was maintained lowering the possibility to include large blasts [9].

### 3.5. Cytokine Quantification

The IL-10 quantification was performed in supernatants of PBMC cultures using the FluoCytomix Analyte Detection kit (eBioscience: San Diego, CA, USA) according to the manufacturer’s instructions.

### 3.6. Statistical Analysis

A Fisher’s exact test was computed for 2 × 2 tables as the first step for analyzing all data sets related to healthy control and T1D populations. The paired *t* test was used to compare unstimulated *versus* stimulated cells in the two groups. The normal distribution of values for healthy control and T1D populations was tested using the Kolmogorov-Smirnov test. The two-tailed Student *t* test was used to compare healthy control and T1D subjects; the unpaired *t* test with Welch’s correction was used when the KS test was not statistically significant, while the Mann-Whitney test was used when the KS test was statistically significant. The results were analyzed using GraphPad Prism software version number 5.00 (GraphPad Software: San Diego, CA, USA). A result with *p* < 0.05 was considered statistically significant.

## 4. Conclusions

In conclusion, this pilot study suggests a defective activation of Tregs in long-standing diabetics due to a lower expression of PD-1 on their surface. These results encourage the performance of further studies on the role of the PD-1/PD-L1 pathway in controlling the autoimmune process in T1D patients for the possible development of novel therapeutic treatments through its modulation.

## Supplementary Materials

Supplementary materials can be found at http://www.mdpi.com/1422-0067/16/09/22584/s1.
