# Supplementary material for: Expression of PD-1 Molecule on Regulatory T Lymphocytes in Patients with Insulin-Dependent Diabetes Mellitus
Source: Int J Mol Sci. 2015 Sep 18;16(9):22584–605. doi: 10.3390/ijms160922584 (PMC4613325; doi:10.3390/ijms160922584)
Supplement: Supplementary file 1 [file ijms-16-22584-s001.pdf]

Supplementary Information

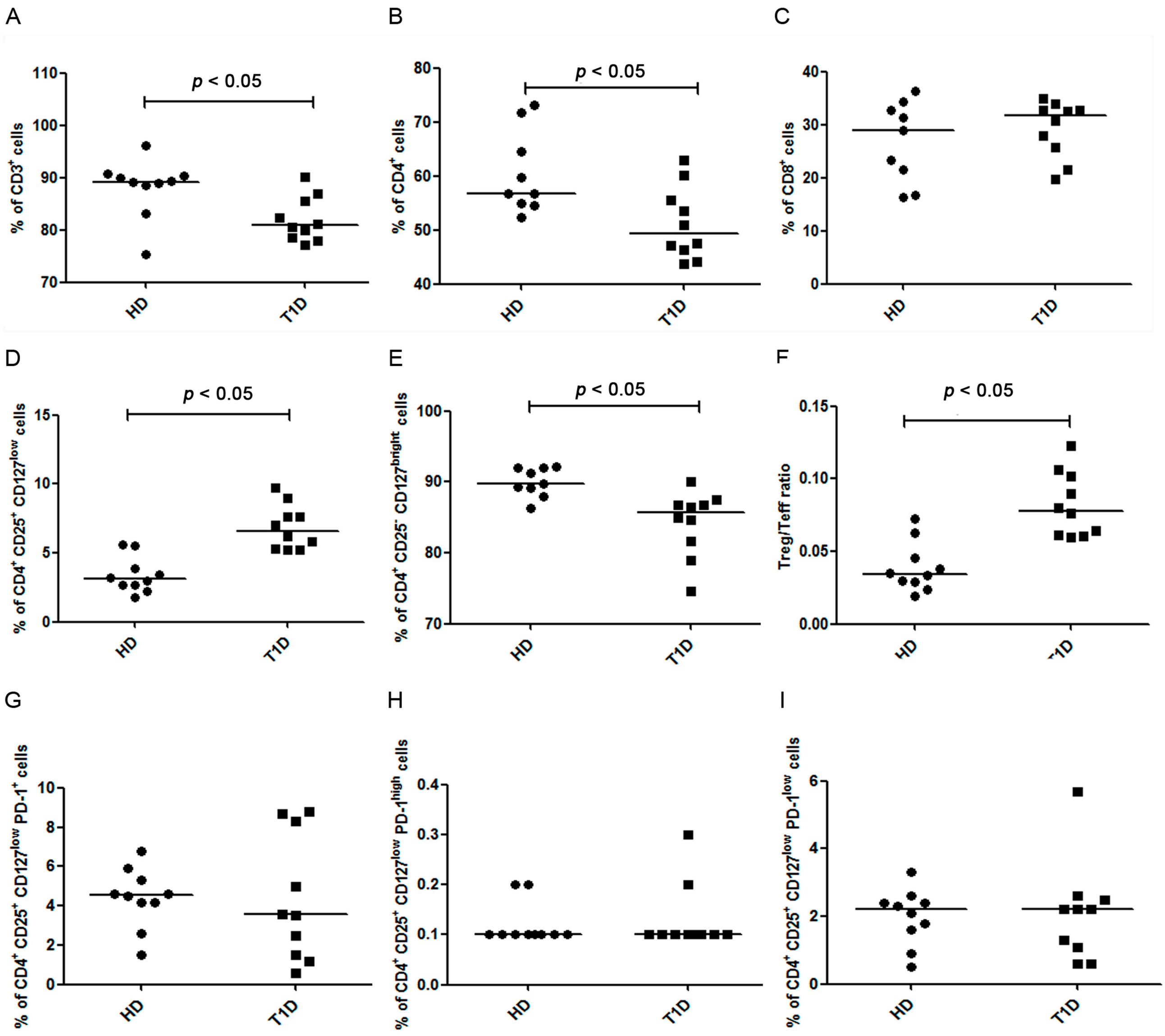

Figure S1. T cell phenotype and percentages of PD-1$^+$ Tregs in healthy controls and in T1D patients after 4 days of culture under standard basal conditions. The graphs show the same analysis of Figure 1 with horizontal lines representing the median frequency. The relative frequency of CD3$^+$ (**A**); CD4$^+$ (**B**); CD8$^+$ (**C**); Treg (**D**); Teff (**E**) cells and the ratio of Treg and Teff percentages (**F**) is shown; Graphs (**G–I**) show the frequency after four days of culture of total PD-1$^+$, PD-1^{high} and PD-1^{low} Treg subfractions respectively in healthy controls and T1D patients. The representation with the median did not considerably change the previous results plotted in Figure 1.

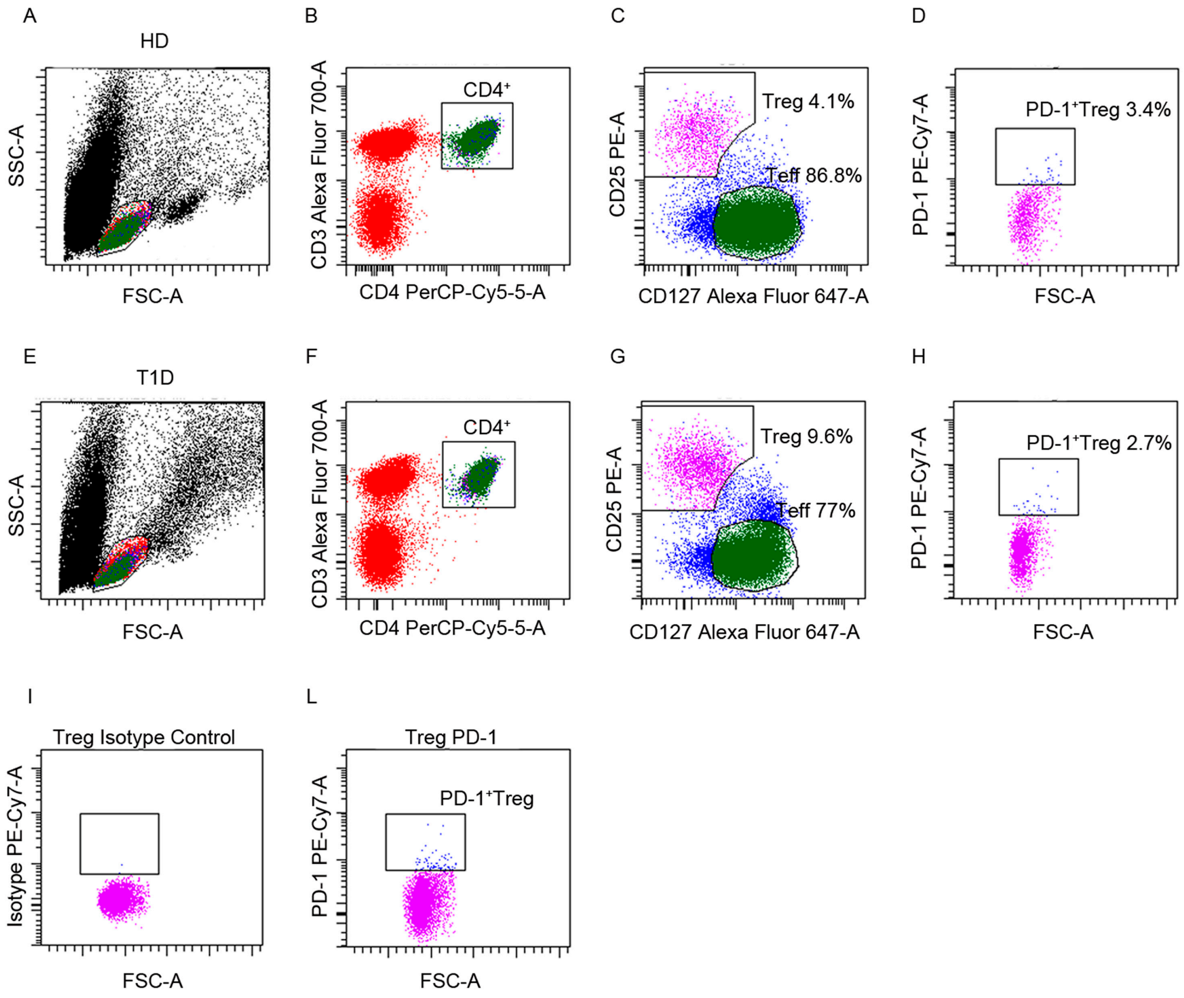

Figure S2. FACS gating strategy to analyze Tregs and their PD-1 expression under standard basal conditions. Treg analysis in the PBMC of a healthy control group (**A**–**D**) and of a T1D patient group (**E**–**H**) after 4 days of culture. Panels **A** and **E** represent examples of FSC-A versus SSC-A dot plot profiles used to define the lymphocyte gate on (**B**,**F**). Representative dot plot analysis showing the expression of CD3 and CD4 on peripheral blood cells gated on lymphocytes; $CD3^+CD4^+$ population defines the gate for $CD4^+$ cells used in panels **C** and **G** to identify regulatory T cells (Tregs, pink dots) ($CD4^+/CD25^+/CD127^{low}$) and effector T cells (Teffs, green dots) ($CD4^+/CD25^-/CD127^{high}$); Treg and Teff frequency in $CD4^+$ T cells are indicated (**C**,**G**). Representative dot plot analysis of the gated Treg population showing percentages of PD-1^+ Tregs (blue dots) are shown in (**D**,**H**). Graphs **I** and **L** show dot plot analysis of the negative reactivity obtained within the Treg gate with the anti IgG1 (k chain) PE-Cy7 conjugated isotope control (**I**) (1:50 dilution, BD Biosciences) and the reactivity of the PD-1 PE-Cy7 (eBioscience) antibody used in our studies.

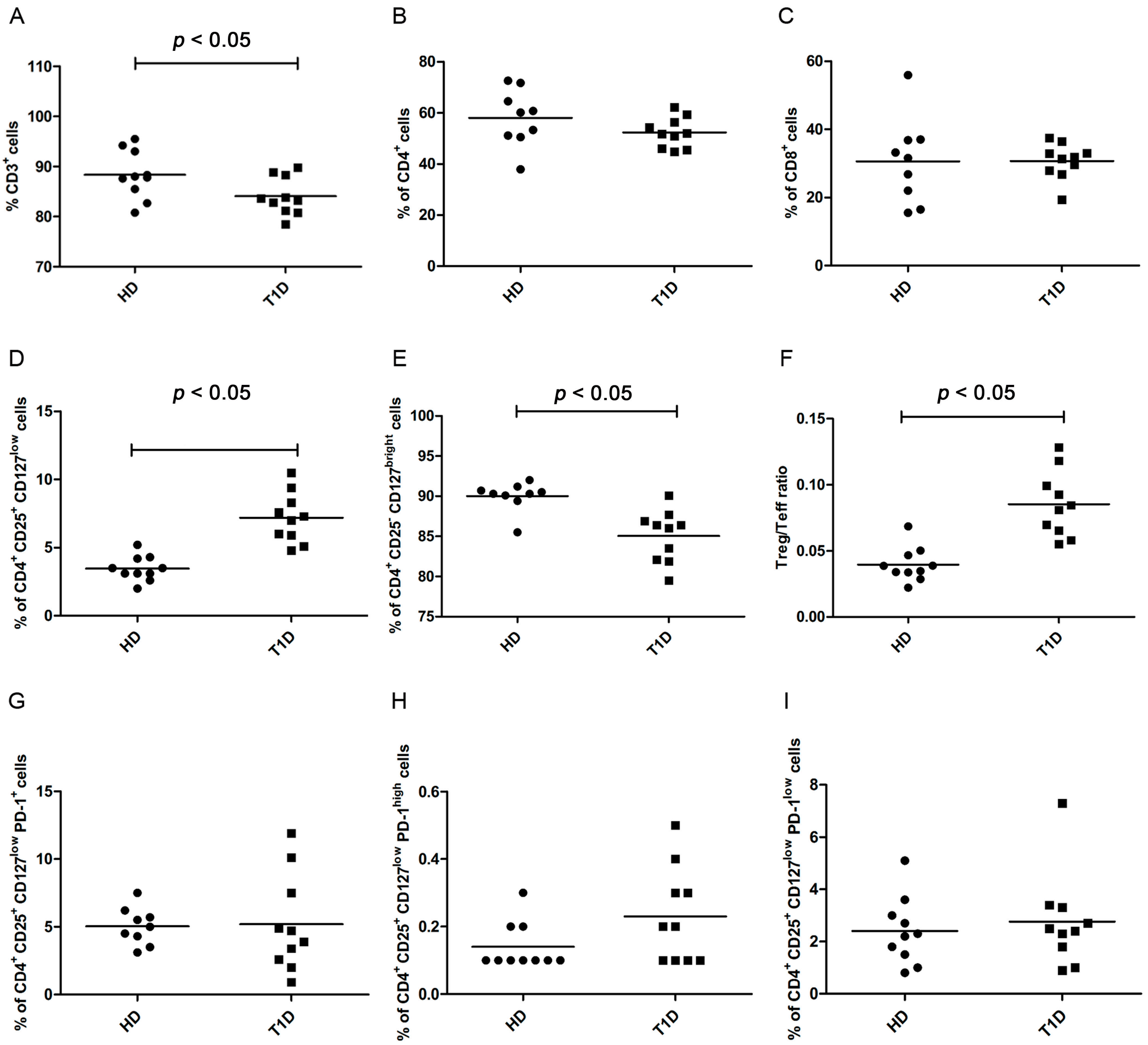

Figure S3. T cell phenotype and percentages of PD-1$^+$ Tregs in healthy controls and T1D patients after 6 days culture under standard basal conditions. Graphs (**A**–**F**) show the same analysis shown in Figure 1; Graphs (**G**–**I**) show the frequency after 6 days of culture of total PD-1$^+$, PD-1^{high} and PD-1^{low} subfractions, respectively, in healthy controls and T1D patients. Frequencies refer to analyzed events within flow-cytometry gates as shown in representative dot plots in Figure S1.

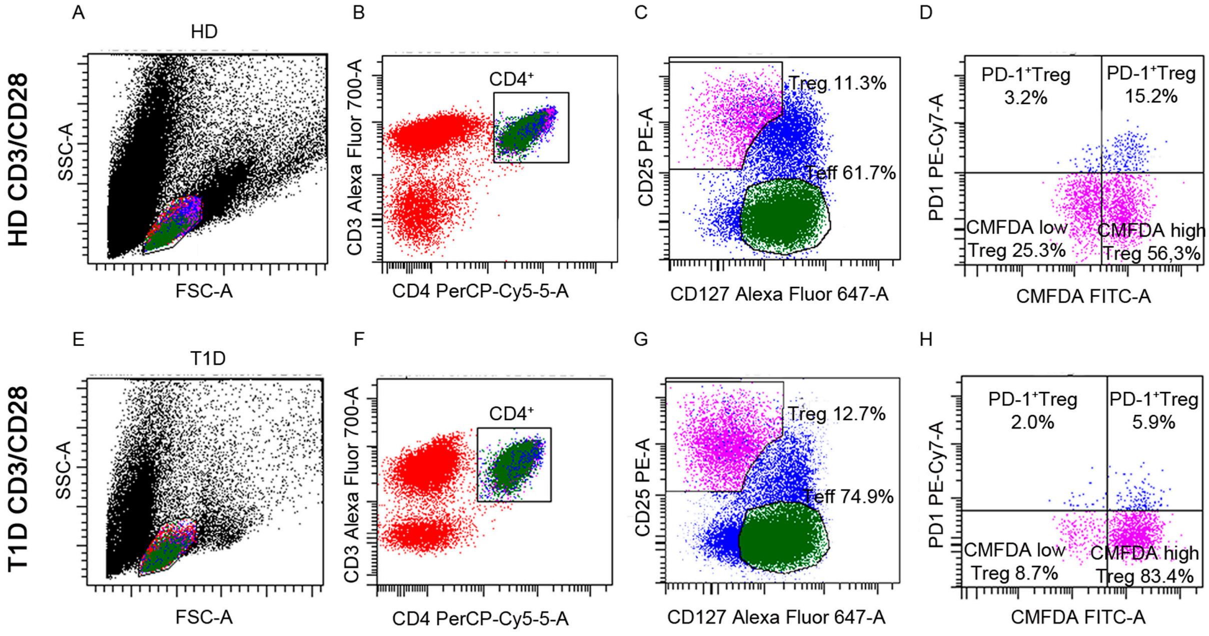

Figure S4. FACS gating strategy to analyze Treg proliferation and Treg PD-1 expression after 4 days of CD3/CD28 stimulation. FACS gating strategy to analyze the Treg proliferation and PD1^{+} Treg cells in healthy controls (**A**–**D**) and T1D patients (**E**–**H**) after 4 days of CD3/CD28 stimulation. Panels **A** and **E** show representative examples of FSC-A versus SSC-A dot plot profiles used to define the lymphocyte gate on (**B**,**F**). Representative dot plot analysis showing the expression of CD3 and CD4 (**B**,**F**) on peripheral blood cells gated on lymphocytes; CD3^{+}CD4^{+} population defines the gate for CD4^{+} cells used in panels **C** and **G** to identify Tregs (pink dots) (CD4^{+}/CD25^{+}/CD127^{low}) and Teffs (green dots) (CD4^{+}/CD25^{-}/CD127^{bright}); Treg and Teff frequency in CD4^{+} T cells are indicated (**C**,**G**). Graphs **D** and **H** show the Treg proliferative response and the Treg PD-1 expression (blue dots) after 4 days of CD3/CD28 stimulation. The gate for Treg cells (**C**,**G**) was defined on CMFDA and PD-1 expression. The frequency of PD-1^{+} Tregs, CMFDA^{low} positive cells and PD-1^{+} Tregs (CMFDA^{low}) are shown in a representative healthy control (**D**) and T1D patient (**H**).

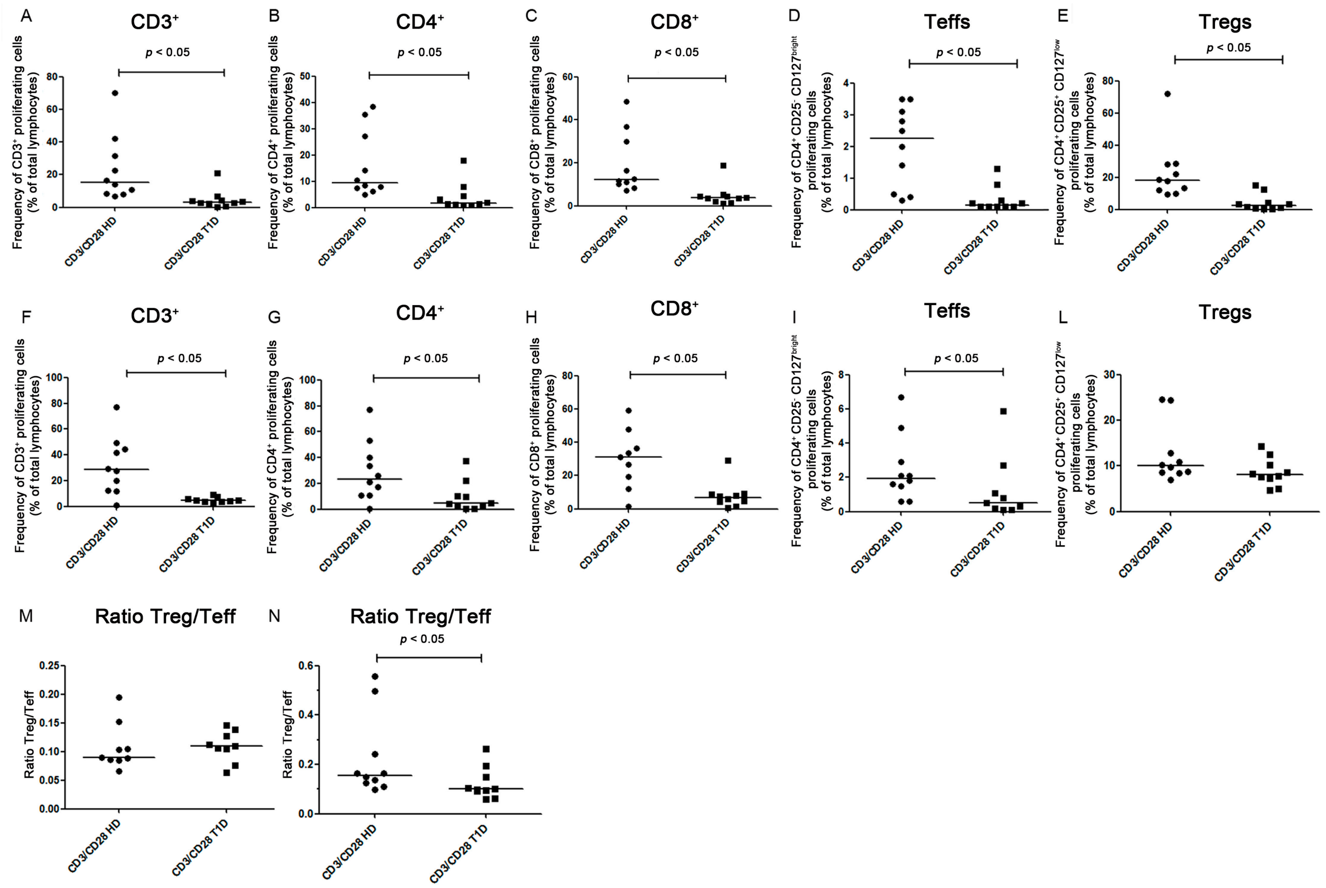

Figure S5. T cell, Teff, Treg proliferative responses and the Treg/Teff ratio after CD3/CD28 stimulation in healthy controls and in T1D patients. The graphs show the same analysis of Figure 2 with horizontal lines representing the median frequency. Graphs show the frequency of $CD3^+$, $CD4^+$, $CD8^+$, Teff, Treg proliferating cells after 4 (**A**–**E**) and 6 days (**F**–**I**,**L**) and Treg/Teff ratio after four days (**M**) and six days (**N**) of culture in healthy controls and T1D patients. The representation with the median did not considerably change the previous results plotted in Figure 2.

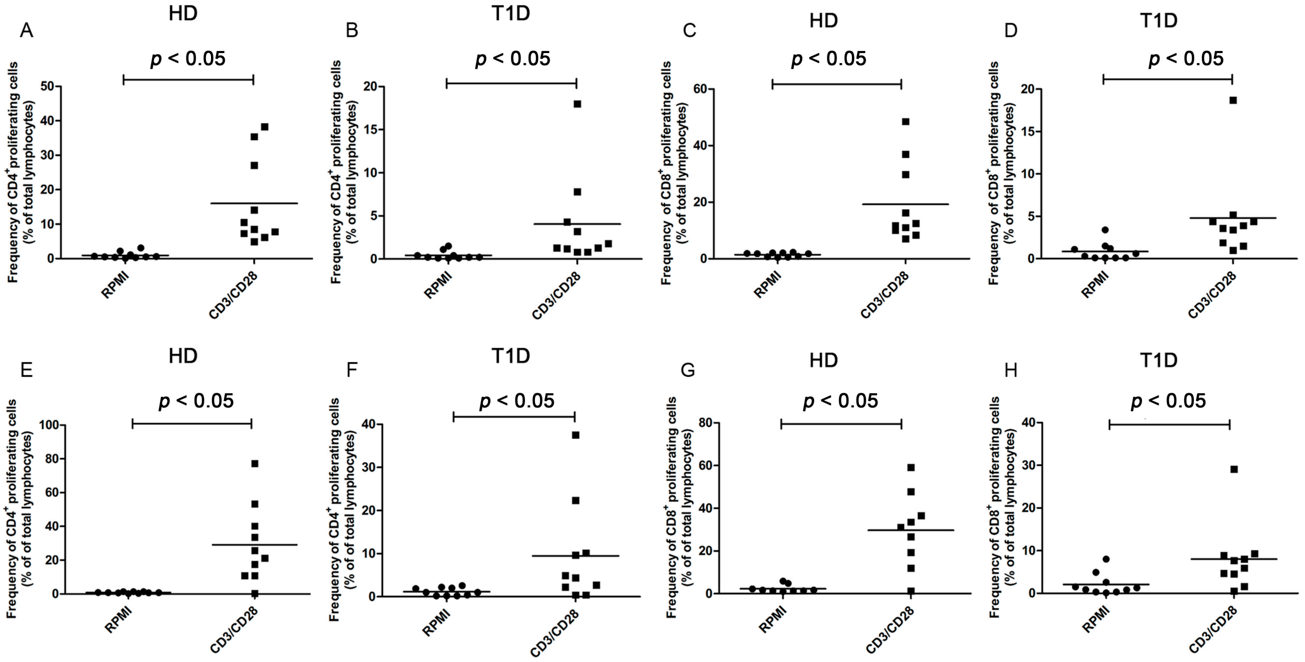

Figure S6. Proliferation of CD4$^+$ and CD8$^+$ T cells after CD3/CD28 stimulation in healthy controls and in T1D patients. Proliferative response of CD4$^+$ after 4 days (**upper** panel) and 6 days (**bottom** panel) in healthy controls (**A**,**E**) and in T1D patients (**B**,**F**). The same proliferative responses of CD8$^+$ T cells after 4 days (**upper** panel) and 6 days (**bottom** panel) are shown for healthy controls (**C**,**G**) and for T1D patients (**D**,**H**). The proliferative response was calculated as the frequency of CMFDA^{low} CD4$^+$ and CD3$^+$CD4$^-$ cells inside the lymphocyte gate.

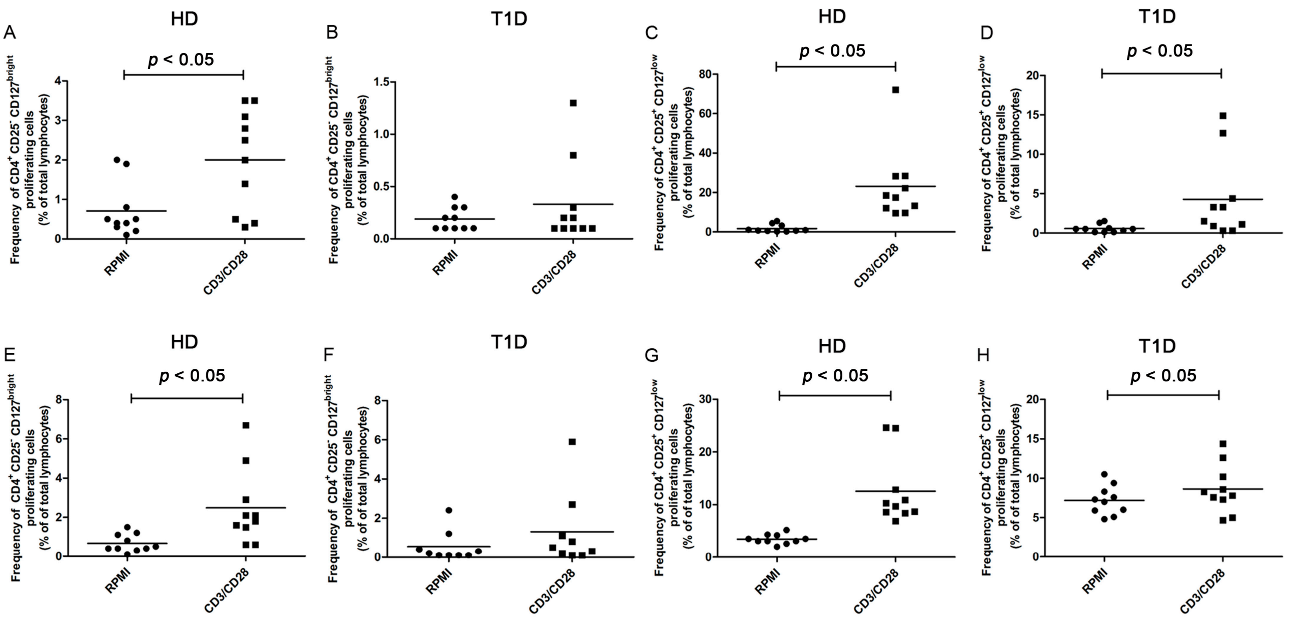

Figure S7. Proliferation of Teffs and Tregs in healthy controls and in T1D patients. Proliferative response of Teffs after 4 days (**upper** panel) and 6 days (**bottom** panel) in healthy controls (**A**,**E**) and in T1D patients (**B**,**F**) and of Tregs after 4 days (**upper** panel) and 6 days (**bottom** panel) of CD3/CD28 stimulation in healthy controls (**C**,**G**) and in T1D patients (**D**,**H**) calculated as described above.

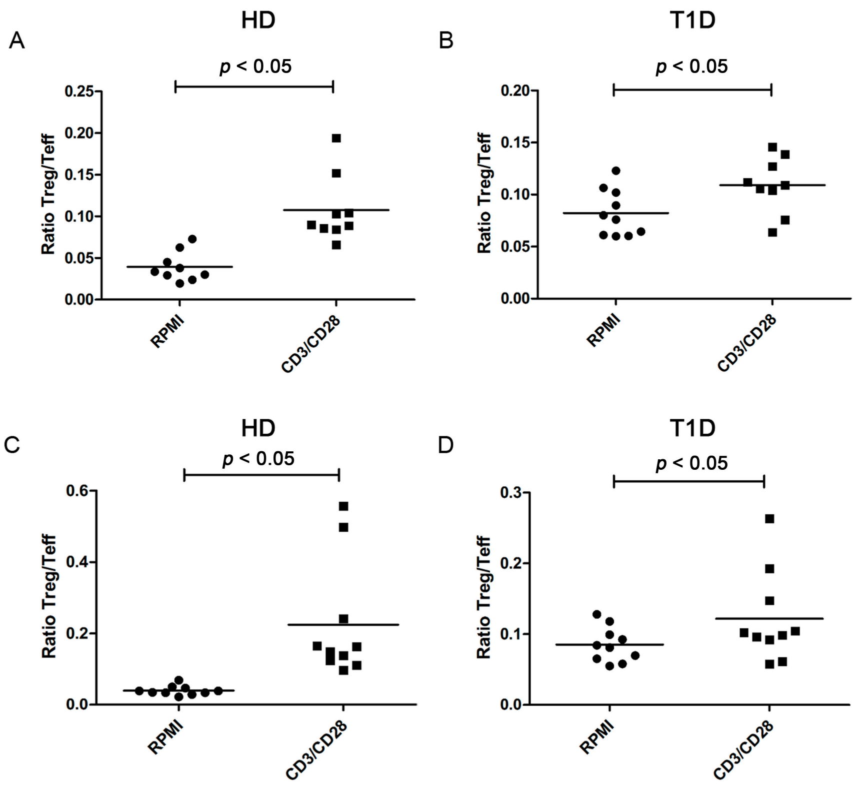

Figure S8. Treg/Teff ratio in healthy controls and in T1D patients. Treg/Teff ratio after 4 days (**upper** panel) and 6 days (**bottom** panel) of CD3/CD28 stimulation in healthy controls (**A**,**C**) and in T1D patients (**B**,**D**).

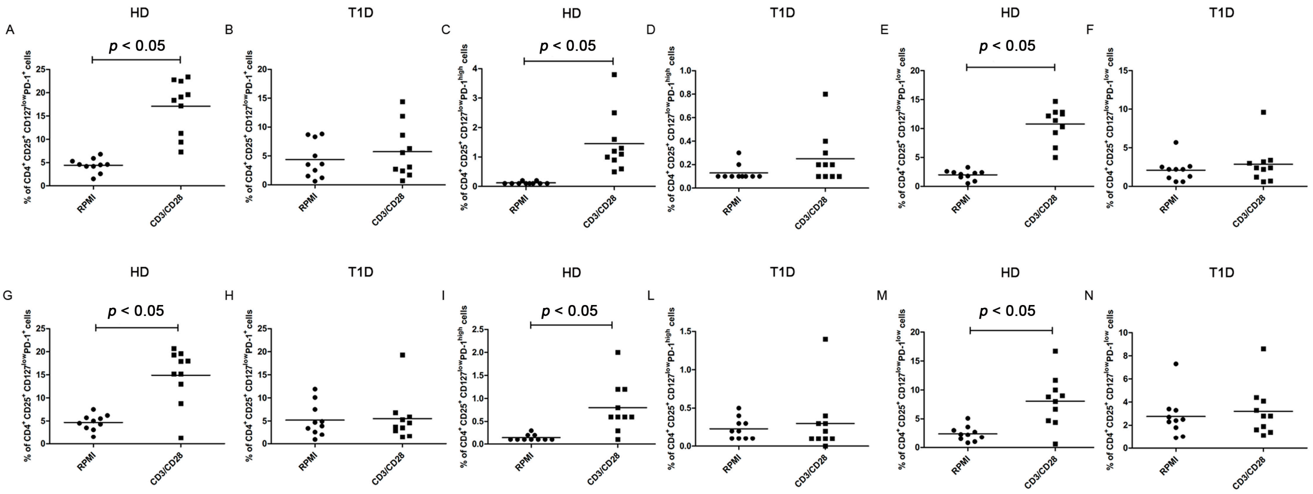

Figure S9. Frequency of total PD-1^{+}, PD-1^{high} and PD-1^{low} Tregs after CD3/CD28 stimulation. Frequency of PD-1^{+} Tregs in healthy controls (**A**,**G**) and in T1D patients (**B**,**H**), of PD-1^{high} in healthy controls (**C**,**I**) and in T1D patients (**D**,**L**) and PD-1^{low} Tregs in healthy controls (**E**,**M**) and in T1D patients (**F**,**N**) after 4 days (**upper** panel) and 6 days (**bottom** panel) of stimulation.

Table S1. Correlation between immune cell subsets and HbA1c values.

Immune Cell Subtypes	Immune Cell Subtype Definition	4 Days HbA1c r	6 Days HbA1c r
$CD3^+$ T cells	% of lymphocytes	0.09	−0.29
$CD4^+$ T cells	% of $CD3^+$ T cells	−0.54	−0.44
$CD8^+$ T cells	% of $CD3^+$ T cells	−0.15	−0.02
$CD4^+$ $CD25^+$ $CD127^{low}$ Treg cells	% of $CD4^+$ T cells	−0.51	−0.52
$CD4^+$ $CD25^-$ $CD127^{bright}$ Teff cells	% of $CD4^+$ T cells	0.35	0.36
Treg/Teff ratio	% of $CD4^+$ $CD25^+$ $CD127^{low}$/ % of $CD4^+$ $CD25^-$ $CD127^{bright}$	−0.50	−0.50
$CD4^+$ $CD25^+$ $CD127^{low}$ $PD1^+$ Treg cells	% of $CD4^+$ $CD25^+$ $CD127^{low}$ Treg cells	−0.32	−0.02
$CD4^+$ $CD25^+$ $CD127^{low}$ $PD1^{high}$ Treg cells	% of $CD4^+$ $CD25^+$ $CD127^{low}$ Treg cells	<u>0.12</u>	0.09
$CD4^+$ $CD25^+$ $CD127^{low}$ $PD1^{low}$ Treg cells	% of $CD4^+$ $CD25^+$ $CD127^{low}$ Treg cells	<u>−0.50</u>	<u>0.29</u>

Data were analyzed after testing normality with Kolmogorov-Smirnov test, then Pearson correlation test or Spearman correlation test (underlined *r* values) were used.
